# Targeted Killing of Monocytes/Macrophages and Myeloid Leukemia Cells with Pro-Apoptotic Peptides

**DOI:** 10.3390/cancers11081088

**Published:** 2019-07-31

**Authors:** Mouldy Sioud, Solveig Pettersen, Ieva Ailte, Yngvar Fløisand

**Affiliations:** 1 Department of Cancer Immunology, Institute for Cancer Research, Oslo University Hospital-Radiumhospitalet, Ullernchausseen 70, N0379 Oslo, Norway; 2 Department of Haematology, Oslo University Hospital-Rikshospitalet, Sognsvannvien 20, N0372 Oslo, Norway

**Keywords:** tumor microenvironment, macrophages, leukemia cells, lytic peptides, targeted therapy, immunotherapy, cancer

## Abstract

Several cells of myeloid origin, such as monocytes and macrophages are involved in various human disorders, including cancer and inflammatory diseases. Hence, they represent attractive therapeutic targets. Here we developed three lytic hybrid peptides, by fusing a monocyte- and macrophage-binding peptide to pro-apoptotic peptides, and investigated their killing potency on blood monocytes, macrophages, and leukemia cells. We first showed that the targeting NW peptide is effective for depleting monocytes from whole peripheral blood mononuclear cells (PBMCs). Incubating the cells with biotin-conjugated NW peptide, and the subsequent capture on streptavidin-conjugated magnetic beads, depleted monocytes from the PBMCs. The NW peptide also depleted myeloid leukemia blasts from patient PBMCs. The treatment of the PBMCs with the lytic hybrid NW-KLA peptide killed monocytes, but not lymphocytes and primary mammary epithelial cells. Additionally, the fusion peptide exhibited a potent toxicity against macrophages and leukemia cells. The free lytic KLA peptide did not affect cells. Similarly, a second lytic hybrid peptide killed macrophages, leukemia cell lines, and blood leukemia blasts from patients with acute and chronic myeloid leukemia. The IC_50_ towards target cells were in the low macromolar range (4–12 µM). Overall, the data indicate that the NW peptide could be a potential drug delivery agent for monocytes, macrophages, and leukemia cells. Moreover, the engineered lytic hybrid peptides acting alone, or in combination with other therapeutic agents, might benefit many cancer patients and overcome drug resistance.

## 1. Introduction

Circulating blood monocytes extravasate into tissues, where they differentiate into M1 or M2 macrophages, controlled by local environmental signals, such as colony-stimulating factor-1 (CSF-1) [1,2]. Under normal conditions, macrophages protect the host against infection and injury and facilitate tissue remodeling [2]. However, in most solid tumors, high macrophage infiltration into tumor tissues has been associated with poor clinical outcome [2,3,4,5,6,7,8,9]. These tumor associated macrophages (TAMs) promote numerous important features of tumor progression, including angiogenesis, motility, metastasis, and inhibition of T cell function [3]. Additionally, TAMs are known to suppress responses to standard-of-care-therapeutics, including chemotherapy, irradiation and angiogenic inhibitors [7,8,9]. In cancer, both the resident and the infiltrating macrophages have the pro-tumorigenic M2 phenotype [4]. Similarly, some studies suggested that elevated number of circulating blood monocytes was associated with poor prognosis in patients with various cancer types [5,7]. In addition to malignancies, macrophages are associated with the progression of a number of other diseases, such as asthma, allergic inflammation, and rheumatic inflammatory diseases [10,11,12,13]. In rheumatoid arthritis (RA), M1 macrophages secrete pro-inflammatory cytokines, such as tumor necrosis factor- (TNF-α) and interleukin-1. The role of these cytokines in RA pathogenesis is well documented by several experimental and clinical findings [11,12]. Hence, therapeutic strategies that either target TAM recruitment from inflammatory monocytes, or deplete TAMs will benefit patients with cancer or inflammatory diseases.

To therapeutically target TAMs, several pharmacological and immunological strategies have been used. Trabecdedin is a synthetic tetrahydroisoquinoline drug originally isolated from the marine Caribbean tunicate *Ecteinascidia turbinate*, approved for the treatment of sarcoma and ovarian carcinoma [14]. In sarcoma patients, trabectedin reduced the density of TAMs and improved patient outcome [15]. Clodronate is a bisphosphonate-family compound found to deplete macrophages, and is currently used to prevent or block the development of bone metastases, as well as to treat inflammatory diseases [16]. In animal models, an antibody targeting the CSF-1 receptor (R) reduced TAMs [17]. Treatment of patients with diffuse-type giant cell tumor with this antibody resulted in clinical benefit that correlated with reduction of TAMs and blood circulating monocytes. Similarly, CSF-1 inhibition by antisense oligonucleotides or small interfering RNAs (siRNAs) suppressed tumor growth in mice xenografted with human cancer cells as a result of macrophages reduction in tumor tissues [18]. Despite these advances, the current therapeutic strategies are not monocyte- and/or TAM-specific, and can thus have substantial side effects over time. Moreover, there is a need for the development of new therapeutic agents to treat drug-resistant cancers.

In recent years, antimicrobial peptides have attracted interest as potential anti-cancer drugs [19,20,21,22,23]. Usually these peptides do not bind to mammalian cell membrane, however, they can kill the cells if internalized via targeting moieties, such as antibodies and peptides [22,23]. Within the cytosol, they selectively disrupt mitochondrial membranes, leading to cell death by apoptosis [20]. Since their killing activity is not dependent on cell proliferation, they do not have many of the undesirable toxic effects of other chemo drugs. In the present study, we evaluated the effects of lytic peptides fused to a targeting peptide on blood monocytes, M1 and M2 macrophages, and leukemia cell lines. In addition, we included blood leukemia blasts from patients with myeloid leukemia, a disease that remains incurable, despite improvement in treatment options. The targeting peptide was identified using phage display libraries [24,25]. Two of the engineered lytic hydrid peptides killed monocytes, macrophages, and leukemia cells in vitro at low peptide concentrations, supporting their further clinical development.

## 2. Materials and Methods

### 2.1. Peptides

The following peptides were purchased from Biosynthesis (Lewisville, TX, USA). Italic letters indicate the pro-apoptotic domains.

NW peptide: NWYLPWLGTNDW-NH_2_
NW peptide-biotin: NWYLPWLGTNDW-GGK-biotin
Control peptide: MEWSLEKGYTIK-GGK-biotin
KLA peptide: *KLAKLAKKLAKLAK*-NH_2_
KLL peptide: *KLLLKLLKKLLKLLKKK*-NH_2_
QLG peptide: *QLGKKKHRRRPSKKRHW*-NH_2_

Fusion lytic peptides:

NW-KLA: NWYLPWLGTNDW**GGG***KLAKLAKKLAKLAK*-NH_2_
NW-KLL: NWYLPWLGTNDW**GGG***KLLLKLLKKLLKLLKKK*-NH_2_
NW-QLG: NWYLPWLGTNDW**GGG***QLGKKKHRRRPSKKRHW*-NH_2_

All peptides were dissolved in sterile water at 600 μM and aliquots and stored at −80 °C until use. Biotin-conjugated NW peptide and control peptide were dissolved in DMSO and stored at −80 °C. A flexible short glycine linker (GGG) was placed between the NW peptide and the lytic domain to minimize potential steric hindrance which may block the binding to target cells.

### 2.2. Antibodies and Cytokines

Cell staining was performed using fluorescein isothiocyanate (FITC), phycoerythrin (PE), allophycocyanin (APC), or pacific blue (PB)-conjugated mouse monoclonal antibodies against CD80, CD86, CD83, CD14, and CD163 (all purchased from BD Biosciences, San Jose, CA, USA). Fluorochrome conjugated antibodies against CD8, CD4, CD56, and CD19 were purchased from BioLegends (Nordic BioSite AS, Kristiansand, Norway). The following cytokines were used: Interleukin-4 (IL-4), granulocyte-colony stimulating factor (GM-CSF), monocyte-colony stimulating factor (M-CSF), tumor necrosis factor-α (TNF-α), interleukin-10 (IL-10), and interferon-γ (IFN-γ; all purchased from R&D Systems (Minneapolis, MN, USA). 

### 2.3. Cell Lines and Peripheral Blood Mononuclear Cells

Leukemia cell lines MV-4-11 and U937 were purchased from the American Type Culture Collection (ATCC, Rockville, MD, USA). Cells were cultured in RPMI medium-1640 supplemented with 10% fetal calf serum (FCS) and antibiotics (complete medium). Primary human mammary epithelial cells were purchased from ATCC. Peripheral blood mononuclear cells (PBMCs) were obtained from buffy coats of healthy individuals and isolated by density gradient centrifugation (Lymphoprep; Nycomed Pharm, Oslo, Norway). Monocytes were prepared using plastic adherence. Briefly, PBMCs were plated into T-75 flasks (3 × 10^6^/mL) in complete RPMI medium and incubated at 37 °C for 1–2 h. Non-adherent cells were removed, and adherent monocytes were harvested by gentle scraping with a plastic cell scraper. CD4+ and CD8+ T cells were isolated using Dynabeads’ positive selection kits (Invitrogen Dynal AS, Oslo, Norway), following the manufacturer’s instructions. CD19+ B cells were isolated by positive selection using CD19 MicroBeads, following the manufacturer’s instructions, using manual labeling and automated separation on autoMACS™ Pro Separator (Miltenyi Biotec, Lund, Sweden). Natural Killer (NK) cells were isolated by negative selection using the NK Cell Isolation Kit (Miltenyi Biotec Norden AB, Lund, Sweden), following the manufacturer’s instructions, in combination with automated separation using autoMACS™ Pro Separator (Miltenyi Biotec Norden AB, Lund, Sweden). The purity of the cells was verified using antibody staining and analysis by flow cytometry.

### 2.4. Primary Leukemia Cells

A single sample of 10 mL of peripheral blood was obtained from each patient with leukemia at Oslo University Hospital. Cells were isolated by density gradient centrifugation as indicated above. All cells were re-suspended in the growth medium, counted, frozen down, or freshly used in downstream experiments. The collection of patient blood samples was approved by the Regional Committees for Medical and Health Research Ethics (REK = 2017/1596). The study was conducted in accordance with the declaration of Helsinki.

### 2.5. Generation of Immature Human Dendritic Cells

Blood monocytes were cultured in complete medium supplemented with IL-4 (100 ng/mL) and GM-CSF (50 ng/mL) for 6 days. Under these conditions, immature DCs are in the supernatant while macrophages stick well to the culture flask. We verified the floating cells to be DCs using flow cytometry detection of CD80, CD86 and CD83. Adherent macrophages are positive for CD14.

### 2.6. Generation of M1 and M2 Macrophages

To generate macrophages, blood monocytes were cultured in X-vivo 15 medium, supplemented with 50 ng/mL GM-CSF (M1) or 50 ng/mL M-CSF (M2), followed by additional two days of culture in the presence of 50 ng/mL LPS and 100 U/mL IFN-γ (M1), or 50 ng/mL IL-4 and 1 ng/mL IL-10 (M2). Under these conditions, the cells showed the morphological characteristic features of M1 or M2 macrophages.

### 2.7. Flow Cytometry

Flow cytometry was performed to analyze the expression of certain surface markers and peptide binding to tested human cells. Conjugated antibodies specific for cell surface markers were incubated with the cells (1–2 × 10^5^ cells/100 μL/sample) in PBS buffer containing 1% FCS or BSA (staining buffer) for 30–60 min at 4 °C. After washing, the cells were re-suspended in 300 μL staining buffer before being analyzed on BD FACS Canto II Flow cytometer, using BD FACSDiva™ software (BD Biosciences, San Jose, CA, USA). Similarly, cells were incubated with biotinylated NW peptide or control peptide (5 μg/mL each) in staining buffer at 4 °C for 40 min. After washing, the cells were incubated with phycoethrin (PE)-conjugated streptavidin, and then processed as indicated above. All data were analyzed by FlowJo software (FlowJo LLC, Ashland, OR, USA).

### 2.8. Depletion of Monocytes and Blast Cells from Peripheral Blood Mononuclear Cells

Peripheral blood mononuclear cells (10^6^ cells) were incubated with biotin-conjugated NW peptide (20 μg/mL) for 40 min at 4 °C with gentle rotation. After washing, streptavidin-magnetic beads were added at a concentration of 4 beads per target cell, and incubation continued for 20 min at 4 °C. The samples were placed on a magnet for 5 min, and non-attached cells were carefully removed and analyzed by flow cytometry to check for the removal of the peptide-binding cells.

### 2.9. Cell Viability Assays

Leukemia cell lines MV-4-11 and U937 were seeded at 10^5^ cells/100 µL complete medium and incubated for 1 h at 37 °C. Cells were subsequently incubated with various concentrations of the different peptides for 1 h at 37 °C. Cell viability was then assessed using CellTiter 96^®^ AQueous One Solution Reagent (Promega, Madison, WI, USA) according to the manufacturer’s instructions. Optical densities were measured at 492 nm. For the M1 and M2 macrophages, monocytes were initially seeded in a 96-well plate (1.5 × 10^5^/100 µL complete medium) and differentiated into M1 or M2 macrophages as described above. After, the culture medium was replaced to remove dead cells and viable adherent cells were treated with various peptide concentrations and processed as above. The effects of the engineered lytic peptides on monocytes and primary leukemia cell viability were measured using propidium iodide (PI) uptake.

### 2.10. Apoptosis Assay

The induction of apoptosis subsequent to peptide treatment was measured using the annexin V/PI staining dual assay, combined with flow cytometry analysis as described previously [22]. Cells stained with annexin alone (green fluorescence) were considered apoptotic, whereas those stained both green and red were considered necrotic.

### 2.11. Uptake of the NW-Peptide Streptavidin-PE-Complexes by Macrophages

Streptavidin-PE conjugates (10 μg/mL) were incubated with biotinylated NW peptide or control peptide (10 μg/mL) for 30 min at room temperature in PBS buffer supplemented with 1% FCS. Then the mixtures were added to macrophages growing in Lab-Tek chamber slides (Nalge Nunc International, Naperville, IL, USA). After incubation for 30 min at 4 °C, the cells were washed 3 times with culture medium and incubated at 37 °C for 60 min to allow internalization of the bound peptide-streptavidin-PE complexes. To visualize the nuclei, Hoechst 33342 (Invitrogen Dynal AS, Oslo, Norway) was added to the cells for 5 min. Subsequently, the cells were washed 3 times with PBS buffer, fixed with 4% paraformaldehyde for 20 min at 4 °C, washed, and then slides were covered with Dako fluorescent mounting medium before analysis by a Zeiss LSM 510 confocal laser scanning microscope (Carl Zeiss, Olympus, Tokyo, Japan). MV-4-11 leukemia cells were analyzed by Zeiss LSM 880 confocal microscope.

### 2.12. Statistical Analysis

All experiments were performed at least three times, except if otherwise indicated. Differences between control and treated cells were measured by the standard student’s *t* test. For multiple comparisons, a two-way ANOVA analysis was used. *p* values < 0.05 were considered significant.

## 3. Results

### 3.1. The NW Peptide Displays Strong Binding to Human Monocytes

**Figure 1:**
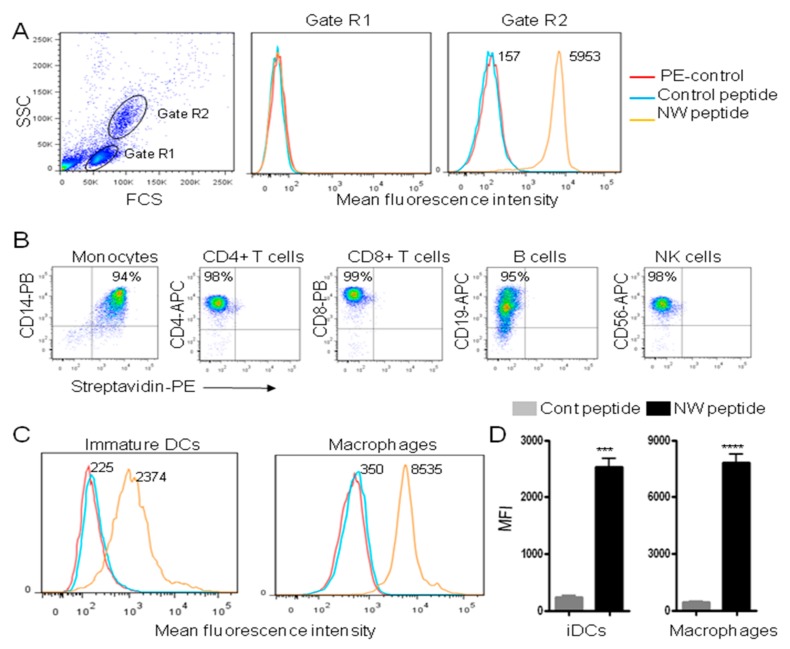
Binding of the NW peptide to blood cells. (**A**) Peripheral blood mononuclear cells (PBMCs) were incubated with the biotinylated W peptide or control peptide (5 μg/mL each) for 40 min at 4 °C. After washing, they were incubated with phycoerythrin (PE)-conjugated streptavidin before analysis by flow cytometry. Gated cells are indicated. The numbers indicate the mean fluorescence intensities (MFI) of the peptide binding. (**B**) Purified blood cell populations were stained with the biotinylated NW peptide in combination with fluorochrome conjugated antibodies specific for CD14, CD4, CD8, CD19, or CD56 cell surface marker, and then analyzed by flow cytometry. The percentages of positive cells are indicated. (**C**) Representative flow cytometry histograms showing the binding of the NW peptide to immature (i) DCs or macrophages. Experimental conditions are as in (**A**). Quantitative data from three independent experiments are shown in (**D**). *** *p* < 0.001, **** *p* < 0.0001.

Unlike standard cancer treatments, targeted therapies are gaining importance, due to their specificity towards cancer cells. Over the last few years, we have developed a panel of peptides that can guide therapeutics to either cancer cells or immune cells [25]. With respect to the latter, we recently identified a peptide (named NW peptide) which binds to monocytes, macrophages and dendritic cells [24]. Figure 1A shows the binding to blood monocyte (gate R2) and lymphocyte (gate R1) populations. The mean fluorescence intensity (MFI) of the peptide binding to monocytes was 38-fold higher than that of the control peptide. By contrast to monocytes, the NW peptide showed no significant binding to the lymphocyte population (T, B, and NK cells).

To further evaluate the specificity of the NW peptide towards blood cells, we analyzed its binding to purified CD14+ monocytes, CD4+ T cells, CD8+ T cells, CD19 B cells, and CD56+ NK cells. The cells were co-stained with the biotinylated NW peptide in combination with cell-lineage specific antibodies (Figure 1B). Under our experimental conditions, only monocytes bound to the NW peptides (first panel). This means that the receptor of the NW peptide is not expressed by cells of lymphoid origin. Immature DCs and macrophages also showed a significant binding to the NW peptide (Figure 1C,D). The binding to macrophages and iDCs had 24 (±2) and 11 (±3) -fold increases over those of the control peptide (*p* < 0.0001 and *p* < 0.001, respectively). Hence, the receptor of the NW peptide seems to be preferentially expressed by monocytes followed by macrophages, and then iDCs.

**Figure 2:**
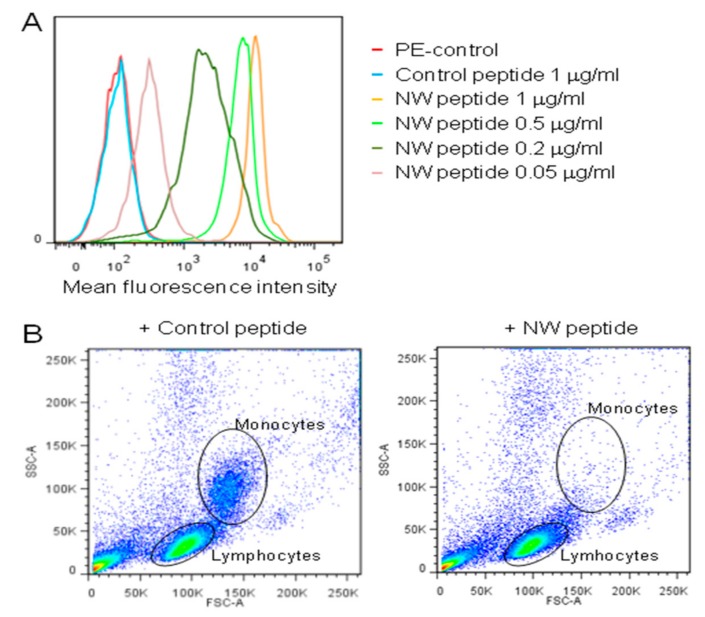
Binding and depletion of blood monocytes. (**A**) Representative flow cytometry histograms showing the peptide binding to purified blood monocytes. Cells were incubated with various concentrations of the biotinylated NW peptide, followed by streptavidin-conjugate PE and analysis by flow cytometry. (**B**) Monocyte depletion. PBMCs were incubated with the biotinylated NW peptide or control peptide (20 μg/mL each), and then processed as described in Material and Methods. The cells were analyzed by flow cytometry to check for monocyte depletion after peptide addition and capture on streptavidin beads. The data are representative of four independent experiments.

Most peptides isolated from phage display libraries have affinities unsuitable for clinical use when synthesized as monomers [25,26]. On the phage, peptides are displayed on the pIII coat protein in five copies at the tip of the filamentous phage particle. As such, peptides selected may bind the cell surface in a multivalent manner [25]. However, the NW peptide exhibited a strong binding to monocytes, even at low peptide concentrations (Figure 2A). This strength of peptide binding is comparable to that of monoclonal antibodies.

Given the high affinity of the NW peptide towards blood monocytes, we next investigated its use in magnetic cell separation protocols. In the majority of such separation techniques, target cells are labeled with magnetic beads that are conjugated to specific antibodies [27]. When different cell populations are placed in a magnetic field, those cells that express the antibody receptor and bind to the beads will be attracted to the magnet, and therefore separated from non-targetted cells. Peripheral blood mononuclear cells were incubated with biotin-conjugated NW peptide for 40 min at 4 °C. After addition of streptavidin-conjugated Dynabeads and magnetic separation, non-attached cells were carefully aspirated off and analyzed by flow cytometry to verify the removal of the monocytes (Figure 2B). The data show that most, if not all, monocytes were depleted from blood PBMCs. Although further optimization is required, recovery of the monocytes from the beads can be done by adding larger excess of competing peptide, thus supporting the use of the NW peptide and derivatives in magnetic separation techniques.

### 3.2. Specific Killing of Monocytes by a Lytic Hybrid Peptide

**Figure 3:**
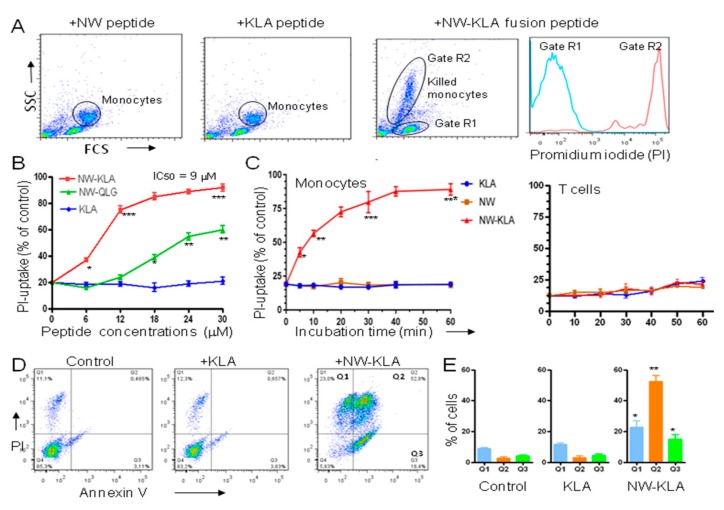
Effects of the fusion peptides on monocyte viability. (**A**) Selective killing of monocytes. Peripheral blood mononuclear cells were incubated with the indicated peptides as described in the text, and then they were analyzed by flow cytometry. Cells were also incubated with propidium iodide (PI) to check for membrane integrity. Data are representative of at least three independent experiments. (**B**) Cytotoxic effects of the fusion peptides. Purified monocytes were cultured in complete medium and then treated for 60 min with various peptide concentrations, incubated with PI and then analyzed by flow cytometry. Data are from three independent experiments. (**C**) Killing kinetics. Purified CD14+ monocytes or CD4+ T cells were incubated with the tested peptides (10 μM each) and then analyzed by flow cytometry at various time points after PI incubation. Data are from three independent experiments. (**D**) Induction of apoptosis. After incubation with the peptides for 60 min at 37 °C, monocytes were analyzed by dual-color flow cytometry for annexin V and PI staining. Quantitative data from three independent experiments are shown in (**E**). * *p* < 0.05, ** *p* < 0.01, *** *p* < 0.001.

Since the NW peptide efficiently depleted monocytes from PBMCs, we generated a peptide fusion with a pro-apoptotic peptide, and investigated the killing potency of the lytic hybrid peptide. For the pro-apoptotic domain, we first selected the cationic -helix peptide (KLAKLAK)_2_ (named KLA peptide), a mitochondrial membrane disrupting agent that has been extensively characterized with respect to structure and killing mechanism [20]. PBMCs were incubated with either NW peptide, KLA peptide, or NW-KLA fusion peptide for 15 min at 4 °C, washed to remove excess of peptides and then incubated at 37 °C for 60 min to allow cell killing. Flow cytometry analysis of cells is shown in Figure 3A. The hybrid lytic peptide killed monocytes, but not lymphocytes, indicating that the killing is specific for monocytes. When the cells were incubated with propidium iodide (PI), only dead monocytes (gate R2) incorporated the dye (Figure 3A, last panel, red histogram). Under the same experimental conditions, the lymphocytes were not killed (gate R1, blue histogram). 

We next compared the cytotoxic effect of the NW-KLA peptide to a second lytic hybrid peptide (named NW-QLG) that has been found to possess a potent cytotoxic effect against breast cancer cell lines, when internalized via a targeting moiety [28]. Unlike the NW-KLA, the NW-QLG peptide did not induce a significant cell killing at lower peptide concentrations (Figure 3B). The IC_50_ values for the two peptides were 9 M and >30 M, respectively. As shown in Figure 3C, exposing monocytes to the NW-KLA fusion peptide resulted in time-dependent loss of cell viability. Most of the cells were killed within a 10–15 min incubation time. Purified T cells were used as a control, as they did not bind to the NW peptide. Under the same experimental conditions, T cells were not killed (Figure 3C).

Usually, cells expose on their surface phosphatidylserine (PS) when they undergo apoptosis [29]. Annexin V binds to PS and therefore can be used to monitor this early event in apoptosis process. Hence, we performed a flow cytometry analysis to determine the death pathway of the targeted lytic peptide using a Dead Cell Apoptosis Kit with Annexin V-FITC and PI. In these experiments, purified monocytes were used. Treatment of the cells with the NW-KLA peptide caused an increase in annexin V positive cells (Figure 3D, a representative example). More than half of the cells (52.8%, ± 10%, *p* < 0.01) were at late apoptosis/secondary necrosis phases (Annexin V+ and PI+) (Figure 3E). Although PS may be exposed under a variety of circumstances and could be a general indicator of membrane instability [30,31,32], the data suggest that monocytes undergo both apoptosis and necrosis pathways after treatment with the NW-KLA peptide. Again, the KLA peptide showed no significant effect as compared to untreated cells.

### 3.3. Effects of the Fusion Lytic Peptides on M1 and M2 Macrophages

**Figure 4:**
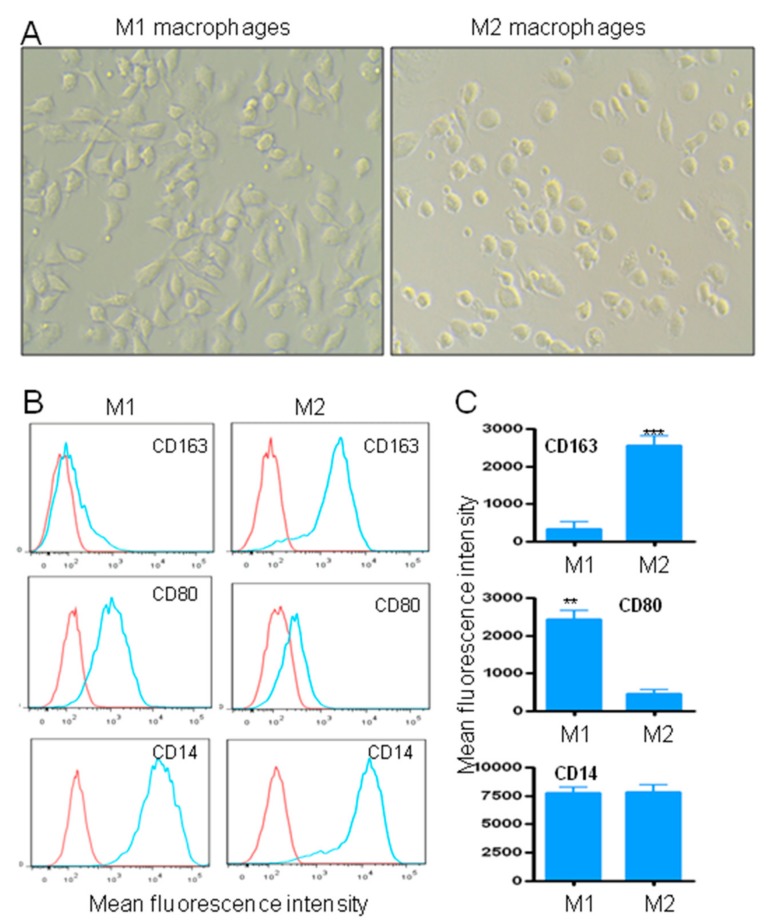
Monocyte-derived M1 and M2 macrophages. (**A**) Morphology of monocyte-derived M1 or M2 macrophages in X-vivo 15 medium. Original magnification, x20. (**B**) Phenotypic characterization of M1 and M2 macrophages. Cell surface expression of CD163, CD80 and CD14 markers by M1 and M2 macrophages. (**C**) Quantitative data from three independent experiments are presented as a mean ± SD. ** *p* < 0.01, *** *p* < 0.001.

One strategy to deplete TAMs is to cut off their replenishment, by circulating inflammatory monocytes. Hence, the killing of blood monocytes, as demonstrated in Figure 3, should result in reduced numbers of TAMs in primary and metastatic sites. As M2 TAMs promote cancer progression and resistance to therapy, we investigated whether the engineered targeted lytic peptides could preferentially kill M2 or M1 macrophages. Monocyte polarization into M1 or M2 macrophages was induced in vitro as described in Materials and Methods. In accordance to previous studies [33], M1 and M2 cells presented either an elongated (M1) or round (M2) morphology, respectively (Figure 4A). The M2 phenotype was further characterized by analyzing the expression of the specific marker CD163. At day eight after differentiation, the expression of CD163 by M2 was significantly increased when compared to M1 macrophages (Figure 4B,C, *p* < 0.001). In contrast to M2, M1 macrophages are characterized by the expression of CD80 and the absence of CD163 [34]. In this respect, the expression of CD80 on M1 macrophages was significantly upregulated, as compared to M2 macrophages (Figure 4B,C, *p* < 0.01). As expected, both cell populations expressed the CD14 marker.

**Figure 5:**
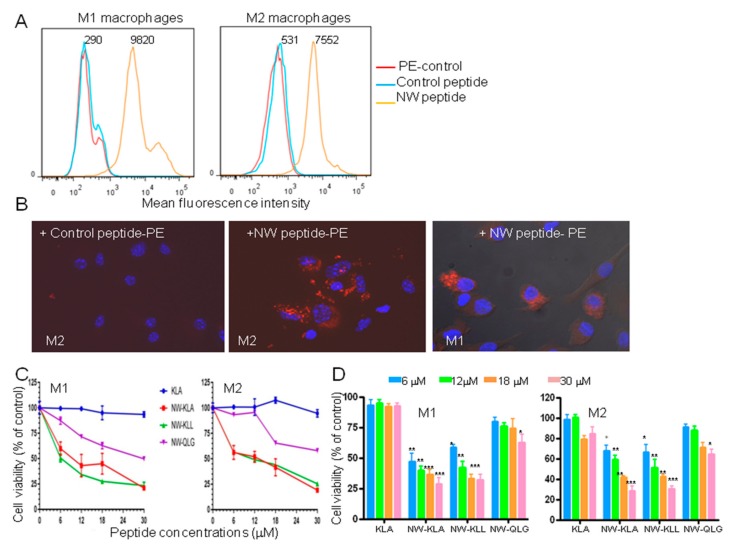
Effects of the fusion lytic peptides on M1 and M2 macrophages. (**A**) Representative flow cytometry histograms showing the binding of the NW peptide to M1 and M2 macrophages. The numbers indicate the mean fluorescence intensities of the peptide binding. (**B**) Internalization of the NW-peptide-streptavidin-PE complexes by M2 and M1 macrophages. Biotinylated NW peptide- or control peptide-streptavidin-PE complexes were added to M2 macrophages and incubated for 40 min at 4 °C. After washing, the cells were incubated at 37 °C for 60 min, further washed, fixed, and then confocal microscopy images were taken. Original magnification, x40. The uptake of the NW peptide-PE complexes by M1 is also shown. (**C**) Cell viability. The cells were incubated with various concentrations of the tested peptides for 60 min at 37 °C, and cell viability was determined using the CellTiter 96R Aqueous One Solution reagent. The results are represented as mean ± SD of triplicate determination. Quantitative data (mean ± SD) from three independent experiments are shown in (**D**). ** *p* < 0.01, *** *p* < 0.001.

Having confirmed the polarization status of the cells, we next tested their binding to the NW peptide (Figure 5A). The peptide bound to both M1 and M2 macrophages. The MFIs of the NW peptide binding to M1 and M2 macrophages were, respectively, 27 and 15 fold higher than those of the control peptide. In most experiments, a small population of M1 macrophages showed a strong binding to the NW peptide. Such binding may contribute to the overall MFI increase.

The potential of using the NW peptide to deliver polypeptides/proteins to macrophages was investigated by examining its ability to promote the internalization of streptavidin-PE conjugates. The cells were pre-incubated for 30 min at 4 °C with preformed biotin-peptide/streptavidin-PE complexes, washed, and subsequently transferred to 37 °C to allow peptide internalization. Since active endocytosis occurs in cells at 37 °C, but not at 4 °C, the complexes can be internalized only after specific binding of the NW peptide to macrophages. Whereas macrophages incubated with the control peptide-PE complexes showed no red fluorescence, those incubated with the NW-peptide-PE-complexes showed intensive cytoplasmic fluorescence resulting from peptide internalization (Figure 5B). It should be noted that streptavidin-PE conjugates did not bind to macrophages (Figure 5A).

In the next experiments, we evaluated the cytotoxic effects of the lytic fusion peptides on macrophages. We included a third lytic domain containing several leucine residues (KLL; KLLLKLLKKLLKLLKKKK) that was shown to induce apoptosis in cancer cells [35]. Although the NW peptide bound better to M1 macrophages when compared to their M2 counterparts, the killing effects of the lytic peptides were comparable (Figure 5C,D). Cells treated with either NW-KLA or NW-KLL lytic peptide were killed effectively when compared to those treated with the other peptides. The IC_50_ values for NW-KLA, NW-KLL and NW-QLG peptides were around 10 ± 2, 6 ± 1, 30 ± 3 μM, for M1 macrophages, and 9 ± 1.5, 9 ± 1.4, >30 μM, for M2 macrophages, respectively. When tested at higher concentrations, the IC_50_ values for the NW-QLG peptide were found to be around 40 μM for M1 and 70 μM for M2 macrophages.

### 3.4. Effects of the Fusion Lytic Peptides on Leukemia Cell Lines 

**Figure 6:**
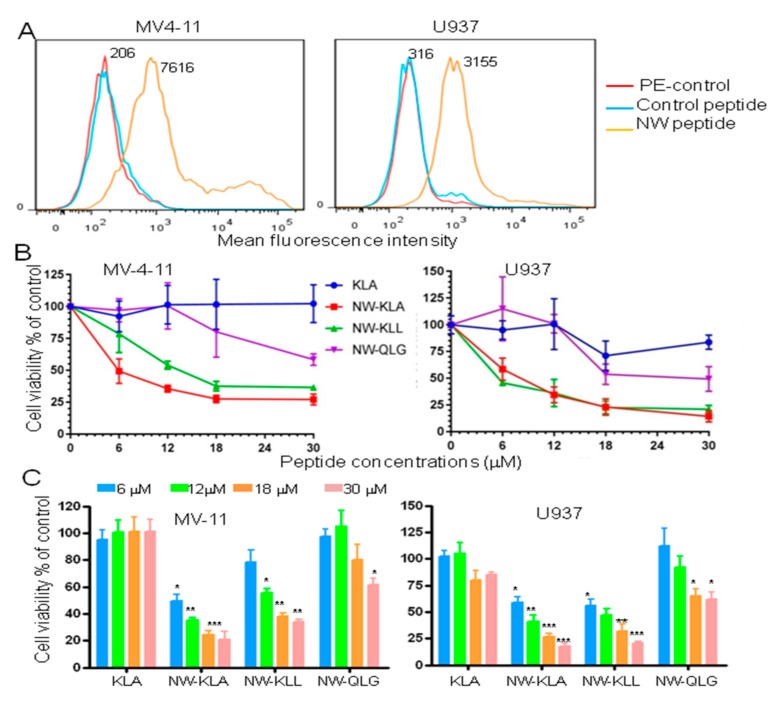
Effects of the fusion lytic peptides on human leukemia cell lines. (**A**) Representative flow cytometry histograms showing the binding of the NW peptide to MV-4-11 and U937 leukemia cells. The numbers indicate the mean fluorescence intensities of the peptide binding. (**B**) Cell viability in response to the peptides. The cells were incubated with various peptide concentrations for 60 min at 37 °C, and then cell viability was determined using the CellTiter 96R Aqueous One Solution reagent. The results are represented as mean ± SD of triplicate determination. Quantitative data (mean ± SD) from three independent experiments are shown in (**C**). * *p* < 0.05, ** *p* < 0.01, *** *p* < 0.001.

Although many advances have occurred in the treatment of blood malignancies, the treatment of relapsed/refractory acute myeloid leukemia (AML) remains one of the most challenging tasks in oncology today [36,37]. Hence, novel treatment strategies are needed. We first tested the binding of the NW peptide to MV-4-11, a human acute myeloid leukemia cell line, and to U937, a pro-monocytic human myeloid leukemia cell line. As shown in Figure 6A, the NW peptide bound to both cell lines. Cells incubated with the lytic hybrid NW-KLA or the NW-KLL peptide showed significant toxicity when compared to those incubated with free KLA or NW-QLG peptide (Figure 6B). In the case of U937 cells, both NW-KLA and NW-KLL peptides had comparable IC_50_ values (5–8 µM). MV-4-11 cells seem to be more sensitive to the NW-KLA peptide (IC_50_ = 4 µM) than the NW-KLL peptide (IC_50_ =12 µM). Again, the pro-apoptosis QLG domain did not induce killing of leukemia cell lines at lower concentrations (<12 µM). At higher concentrations, the NW-QLG IC_50_ values for MV4 and U937 were around 60 and 90 µM, respectively.

**Figure 7:**
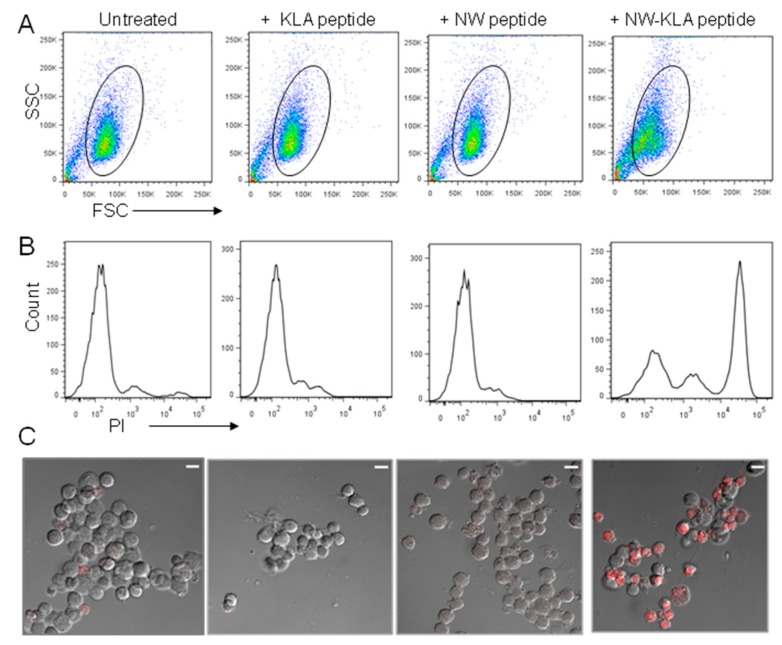
Permeability of MV-4-11 cells after treatment with the NW-KLA peptide. (**A**,**B**) MV-4-11 cells were treated with the test peptides (6 μM each) for 20 min at 37 °C, incubated with PI and then analyzed by flow cytometry. (**C**) Confocal microscopy images. After incubation with PI, the cells were washed and resuspended in 100 μL PBS buffer. One drop of each cell suspension was spotted onto microscope glass slips and processed for confocal microscopy. Images are of single sections through the middle of cells. Scale bar represents 10 μm. The same samples shown in A were analyzed in B and C.

We next evaluated the permeability of MV-4-11 cells to promidium iodide (PI) after peptide treatment. PI does not stain live cells, due to the presence of an intact plasma membrane. As indicated by the forward scatter (Figure 7A), cells treated with the NW-KLA peptide for 20 min showed a smaller size and most cells were porous to PI (Figure 7B, last panel), indicating that they were killed. In contrast, cells treated with the control peptides remained unchanged compared to untreated cells. In accordance with the flow data, confocal microscopy images showed that the cells treated with the NW-KLA peptide were permeable to PI, making them become red.

### 3.5. Effects of the Lytic Peptides on Primary Leukemia Cells

**Figure 8:**
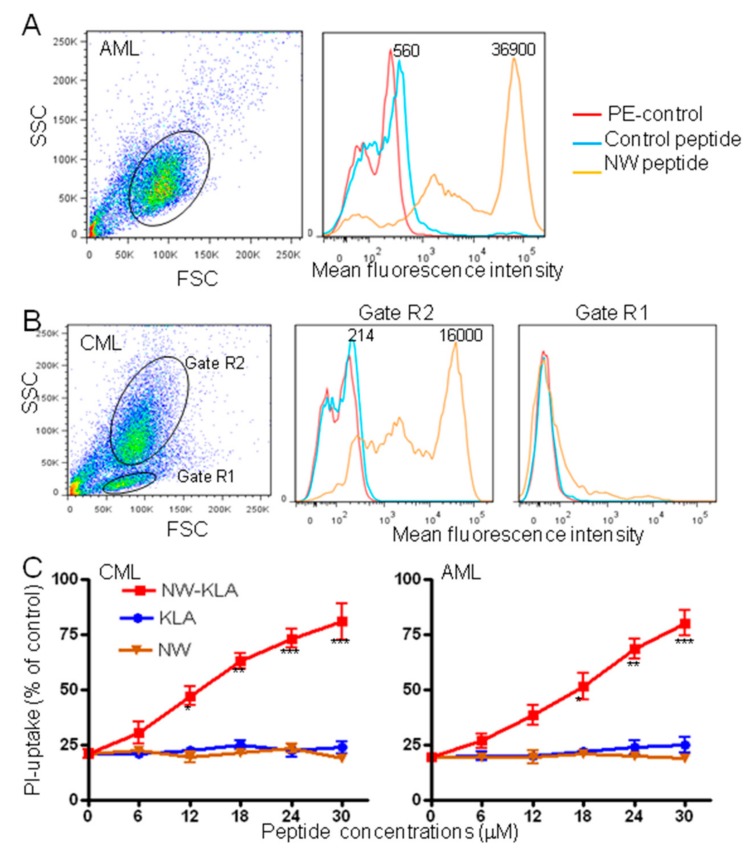
Effects of the targeted lytic peptides on human primary leukemia cells. Representative flow cytometry histograms showing the binding of the NW peptide to primary leukemia cells from a patient with acute myeloid leukemia (AML) (**A**) or from a patient with chronic myeloid leukemia CML (**B**). Gated cells are indicated. The numbers indicate the mean fluorescence intensities of the peptide binding (**C**). Cell viability in response to the peptide treatment. The cells were incubated with various concentrations of the tested peptides for 60 min at 37 °C, and then cell viability was determined using flow cytometry subsequent to PI incubation. The results are represented as mean ± SD of three independent experiments. * *p* < 0.05, ** *p* < 0.01, *** *p* < 0.001.

**Figure 9:**
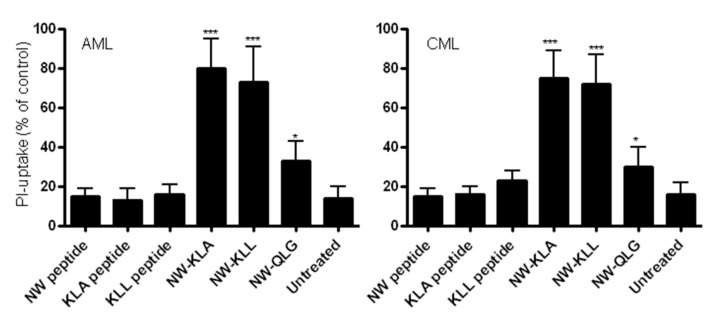
Cytotoxic effects of the fusion peptides on primary leukemia cells. Cells were incubated with the indicated test peptides (12 µM each) for 120 min at 37 °C followed with PI staining and analysis by flow cytometry. The data are from three independent experiments and are presented as mean ± SD. * *p* < 0.05, *** *p* < 0.001.

Although the life expectancy of patients with acute myeloid leukemia (AML) or chronic myeloid leukemia (CML) has improved in recent years, treatment resistant and refractory leukemia are still a major problem [36,37]. Hence, novel approaches to treatment are urgently needed to further improve the prognosis of these diseases. Prompted by the strong cytotoxic effects of the engineered lytic hybrid peptides on leukemia cell lines, we investigated next, whether the NW-KLA peptide would kill primary leukemia cells. Figure 8 shows the binding of the NW peptide and control peptide to freshly isolated blood AML (Figure 7A) and CML (Figure 7B) blasts. By contrast to the control peptide, the NW peptide showed a very strong binding to both blast types, indicating that the peptide receptor is overexpressed in primary leukemia cells when compared to leukemia cell lines and macrophages. In the case of the CML, the lymphocyte population (gate R1) did not bind to the NW peptide, again arguing that the peptide binds to a receptor that is expressed by cells of myeloid, but not lymphoid origin. Treatment with the NW-KLA peptide induced cell death in a dose-dependent manner (Figure 8C). When tested at 10 μM for 2 h, the lytic hybrid peptides showed significant cytotoxic effects on blast cells (Figure 9). The average decrease in viability of freshly isolated AML blasts due to the treatment with the NW-KLA and NW-KLL lytic peptides were 88 ± 5% (*p* < 0.001), 75 ± 6% (*p* < 0.001), respectively. Similarly, these two lytic peptides induced around 74 ± 7% of cell death in CML blasts (*p* < 0.001). There was no significant difference in viability between untreated cells and those treated with the control peptides. Comparable results were obtained with additional three patients with AML and four patients with CML.

### 3.6. Depletion of Blood Blast Cells

**Figure 10:**
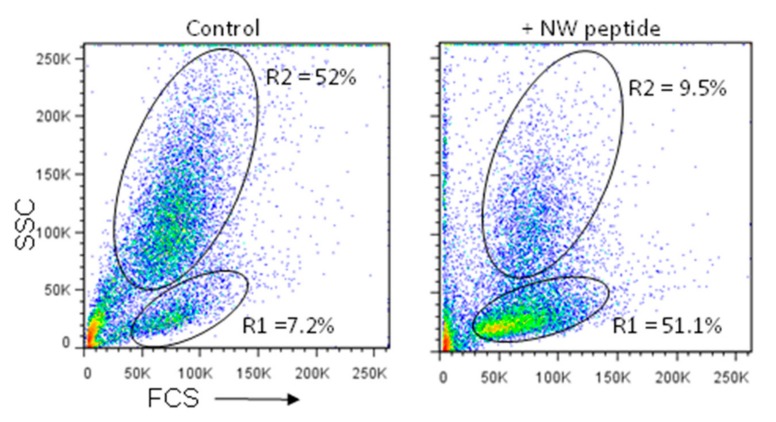
Depletion of blast cells from whole PBMCs. PBMCs from a patient with CML were incubated or not with the biotinylated NW peptide (10 µg/mL), followed by streptavidin-conjugated magnetic beads, as described in Materials and Methods. After magnetic separation, non-binding cells were analyzed by flow cytometry to verify blast depletion. 15,000 events were recorded for each sample.

A number of methods have been used to remove leukemia cells from blood or bone marrow aspirates [38]. In such techniques, the specificity of magnetic affinity separation is based on the selectivity of monoclonal antibody binding to target cells. Given the strong binding of the NW peptide to primary leukemia cells, we investigated whether it can be used to deplete or reduce blast cells from PBMCs. Peptide mediated blast depletion in each PBMC sample was determined by flow cytometry by counting the cells in blast population (gate R2) vs those in lymphocyte population (gate R1) and by relating the ratios with those of the corresponding untreated sample (Figure 10 as a representative example). For both samples, fifteen thousand ungated events were collected. The NW peptide depleted most blood leukemia blasts (9.5% vs. 52%). Notably, the lymphocyte population was significantly enriched (7.2% vs. 51.1%). Thus, the NW peptide can be used to remove blast cells and enrich for other cell types, such as lymphocytes.

### 3.7. Effects of the NW-KLA Peptide on Primary Mammary Epithelial Cells

**Figure 11:**
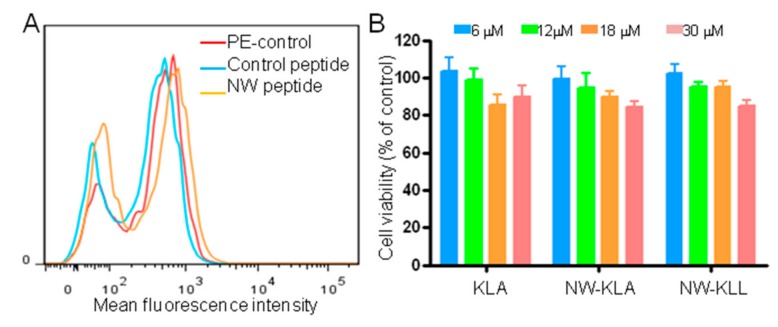
Effects of the fusion lytic peptides on normal mammary epithelial cells. (**A**) Representative flow cytometry histograms showing the binding of the NW peptide to primary human mammary epithelial cells. (**B**) Cell viability. The cells were incubated with various concentrations of the tested peptides for 60 min at 37 °C and cell viability was determined using the CellTiter 96R Aqueous One Solution reagent. The results are represented as mean ± SD of three independent experiments.

Having demonstrated that the lytic hybrid peptides can kill monocytes, macrophages, and leukemia cells, we investigated their cytotoxic effects on primary human mammary epithelial cells (HMECs). We first analyzed the binding of the NW peptide to the cells (Figure 11A). No significant binding was detected, suggesting that the peptide receptor is not expressed by non-hematopoietic cells. Treatment of HMECs by the lytic fusion peptides did not result in significant cytotoxic effects (Figure 11B). Thus, the expression of the peptide receptor on target cells is indispensable for the cytotoxicity of the engineered fusion lytic peptides.

## 4. Discussion

Although abnormalities of cancer genes are essential contributors to cancer, cells within the tumor microenvironment, such as macrophages, myeloid-derived suppressor cells, and T regulatory cells play an important part in the initiation and progression of solid tumors [18,39]. Additionally, host macrophages can promote lymphoma and leukemia cell survival in vitro and in vivo [40,41,42]. By coupling pro-apoptotic peptides to the NW peptide, we engineered new targeted lytic peptides that killed monocytes and macrophages. At the concentrations used, the lytic hybrid peptides did not kill blood lymphocytes. Monocytes and macrophages are widely acknowledged as one of the central suppressive populations within solid tumors, and depleting these cells should benefit patients with solid and blood malignancies. Therapies that deplete TAMs and/or cut off their replenishment by circulating inflammatory monocytes would also benefit patients with various inflammatory diseases.

In several types of cancers, including ovarian, pancreatic, breast, and brain cancers, most, if not all, TAMs express the M2 phenotype [4,5]. In these cancers, specific therapy targeting M1 or M2 macrophages may not be required. Indeed, untargeted depletion of monocytes and macrophages in experimental settings has been successful in inhibiting tumor growth and enhancing responses to standard chemo and anti-angiogenic therapies [43,44]. Recently, Galletti et al. showed that eliminating TAMs along with neutrophils, sensitizes mammary tumors to chemotherapy, resulting in tumor eradication in mice [42]. The authors used a monoclonal antibody to block CSF-1R signaling, required for macrophage development and infiltration into tumor tissues. Similarly, the use of small molecule inhibitors or antisense RNA strategies to inhibit CSF-1R signaling, also inhibited tumor growth in both xenograft and genetically engineered mouse models [45,46,47]. Our current targeting strategy would preferentially eliminate both circulating monocytes and macrophages, and might be better than previously tested strategies. Unlike tissue resident macrophages, which are derived largely from the yolk sac in embryogenesis, TAMs derive from circulating blood monocytes [3]. Using phage display, Cieslewicz et al. selected a peptide specific for murine macrophages [48]. When fused to a pro-apoptotic peptide, the fusion peptide inhibited tumor growth in a subcutaneous tumor model [48]. Unfortunately, the selected peptide showed no significant binding to human M1 and M2 macrophages [48].

With respect to blood malignancies, adults with AML have some of the highest unmet treatment needs of all cancer patients. The outcomes for patients with relapsed or refractory AML are poor, with overall survival estimated at no more than 10% at 3 years [49,50]. In addition to cell lines, the killing potency of the NW-KLA and the NW-KLL fusion peptides was confirmed in leukemia patient samples at low concentrations. In all experiments, the NW-QKG fusion peptide killed leukemia cells only at higher concentrations. Early reports from Ghosh’s group showed that the leucine residues were essential for peptide structure and cytotoxic effect [51]. Most anti-cancer peptides, natural and synthetic, contain several leucine residues. By contrast, the lytic QLG domain contains only one leucine residue, which may explain its weak cytotoxic effects at lower concentrations. Interestingly, the NW-QLG fusion peptide preferentially killed M1 rather than M2 macrophages at high peptide concentrations.

Although promising agents such as Bcr-Abl tyrosine kinase inhibitors have shown a significant therapeutic efficacy in patients with CML [52], the search for innovative therapeutic alternatives in this disease is also essential, due to the emergence of primary or secondary resistance to treatment. Moreover, there is a need in improving the management of CML in blast crisis. A large number of patients with hyperleucytosis can develop leukostasis, a life-threatening situation where leukemia cells are thought to cause organ dysfunction [53]. The lytic hybrid NW-KLA and NW-KLL peptides showed a strong cytotoxic effect against CML blasts. Moreover, we demonstrated that the targeting NW peptide can selectively deplete blast cells from PBMCs. Although in vivo work is needed, the data would support the development of the engineered lytic hybrid peptides as a myeloid cytoreduction therapy.

In addition to solid tumors, macrophages have recently been reported to be involved in tumor progression in several hematological malignancies, such as CML, AML, and B-cell lymphomas [40,41,42,54]. TAMs are highly present in relapsed and refractory lymphomas, most likely playing an important role in multiple types of drug resistance [50]. Recently, Al-Matary et al. showed that myeloid leukemia-associated macrophages can support the progression of AML [40]. The authors showed a significant increase in M2 macrophages in the bone marrow of AML patients when compared to healthy volunteers. These M2 macrophages supported the growth of both human and murine AML cells in vitro. Targeting macrophages using anti-CSF-1R antibody or clodrolip (clodronate encapsulated liposomes) also impaired chronic lymphocytic leukemia cell engraftment. Macrophage depletion sensitized leukemia cells to apoptosis via induction of TNF-α signaling, and leukemia cells were killed through a TNF-α-dependent mechanism [39]. The NW-KLA peptide, unlike other targeted cytotoxins, offers the possibility of targeting monocytes, macrophages, and leukemia cells.

With respect to chemotherapy, cancer cells become resistant to a variety of structurally different drugs, even after treatment with only one single drug [55]. By contrast, lytic peptides, when delivered via a targeting moiety, damage cell membranes within minutes which would hinder formation of resistance. Most lytic peptides killed both drug-sensitive and drug-resistant cancer cells [19,22]. Notably, the tumor selectivity of lytic peptides was considerably enhanced after the fusion to tumor targeting domains, such as antibodies and peptides [20,56]. Moreover, a variety of nanoparticles were also applied for the delivery of lytic peptides, resulting in reduced side effects, and an increase in targeted accumulation in tumor tissues [57,58]. Under our experimental conditions, the KLA peptide did not kill target cells, whereas the hybrid peptides did. Thus, the killing activity requires the presence of the targeting NW domain. Out of the three designed peptides, the NW-KLA and NW-KLL peptides showed the strongest lytic activity. It should be noted that the NW peptide has no effect on cell viability, even at high concentrations.

With respect to peptide selection, a primary advantage of the phage display technology is that affinity-based interactions are detected in native biological systems. The screening on intact cells preserves the original conformation of cell surface proteins and protein-protein interactions that could be relevant in vivo. While a number of selected peptides from phage display libraries have been used as tumor imaging agents, or disease biomarkers without knowledge of their binding receptors [25,59], the clinical use of such peptides will be further facilitated better by the characterization of their binding partners. Moreover, once the partner is known, certain amino acid chains of the peptide can be modified to improve its binding affinity and specificity. Unfortunately, immunoprecipitation experiments with the biotinylated NW peptide failed to identify potential partners. This is most likely due to the intrinsic nature of membrane proteins. They are present in low abundance, and their solubility in most buffers is a major issue. The selection of detergents suitable for the solubilization and purification of a specific membrane protein is critical for the outcome of the experiments. A combination of proteomic and genomic approaches will be needed to tackle this challenging task that is under investigation.

## 5. Conclusions

Macrophages are among the most abundant normal cells in the tumor microenvironment, and usually play a pro-tumoral role. Additionally, they support the proliferation and survival of leukemia and lymphoma cells. By fusing a targeting peptide to pro-apoptotic peptides, we engineered new lytic hybrid peptides that killed monocytes, macrophages and leukemia cells. Killing leukemia cells and macrophages with one single agent, such as the lytic fusion NW-KLA peptide should benefit patients with blood malignancies. In addition to therapy, the NW peptide could be a good candidate for cell depletion from blood and/or bone marrow aspirates.
